# Prediction of Cataract Severity Using Slit Lamp Images from a Portable Smartphone Device: A Pilot Study

**DOI:** 10.3390/s26061954

**Published:** 2026-03-20

**Authors:** David Z. Chen, Changshuo Liu, Junran Wu, Lei Zhu, Beng Chin Ooi

**Affiliations:** 1 Department of Ophthalmology, National University Hospital, Singapore 119024, Singapore; 2 Centre for Innovational and Precision Eye Health, Yong Loo Lin School of Medicine, National University of Singapore, Singapore 119228, Singapore; 3 Department of Computer Science, National University of Singapore, Singapore 117417, Singapore; 4 School of Software Technology, Zhejiang University, Ningbo 315100, China

**Keywords:** cataract, deep learning, screening, teleophthalmology, remote monitoring

## Abstract

Cataract diagnosis requires a comprehensive dilated examination by an ophthalmologist using a slit lamp; there is currently no effective means to objectively screen for cataracts in the community using portable devices without dilation. We hypothesized that it would be possible to predict cataract severity using deep learning on images taken using a portable smartphone-based slit lamp prototype, with and without dilation. In this prospective cross-sectional pilot study, slit lamp images were captured from eligible patients with cataracts in a tertiary clinic using a portable slit lamp prototype attached to a smartphone. The Pentacam nuclear staging score (PNS, Pentacam^®^, Oculus, Inc., Arlington, WA, USA) was taken from the dilated pupils and served as ground truth. A transformer prototypical network with the Swin transformer on the images was trained to assign the class label corresponding to the highest predicted probability. Heat maps were generated based on attribution masks to identify the anatomical areas of concern. A total of 1900 images from 198 eyes of 99 patients were captured. The average age was 65.3 ± 10.4 years (range, 41.0 to 88.0 years) and the average PNS score was 1.57 ± 0.81 (range, 0 to 4). The model achieved an average accuracy of 81.25% and 74.38% for undilated and dilated eyes, respectively. Heat map visualization using the integrated gradient method successfully identified the anatomical area of interest in certain images. This study suggests the possibility of estimating cataract density using a portable smartphone slit lamp device without dilation. Further work is under way to validate this technique in a larger and more diverse group of eyes with cataracts.

## 1. Introduction

A cataract is the result of age-related degeneration of the human crystalline lens. It is the leading cause of reversible blindness worldwide, affecting 35 million people [1]. The current gold standard of cataract assessment is through a slit lamp by an ophthalmologist. There have been several clinical grading scales created for the standardization of this grading, including the Lens Opacities Classification System II (LOCS II) [2], the Lens Opacities Classification System III (LOCS III) [3], the photographic Wisconsin Cataract Grading System [4], and the Oxford Clinical Grading System [5]. Of these, the LOCS III is by far the most popular clinical grading system used in population studies [6].

However, the manual grading of cataracts by trained ophthalmologists is costly and subjective. Multiple studies in the past have attempted to incorporate the use of machine learning to automate nuclear cataract grading [7,8,9,10], including the use of an AI-powered mobile app [11]. In 2015, Gao et al. described a method using a convolutional–recursive neural network to grade nuclear cataracts from standardized images [12]. This method was validated on a population-based dataset of 5378 slit lamp images, achieving a 99.0% decimal grading error of ≤ 1.0. Despite these impressive results, the dataset required dilation, the use of standardized lighting conditions, and photography using a specialized digital slit lamp camera [13]—conditions that are not always available outside of a tertiary setting. Specifically, the need for a bulky photographic device and standardized photographic conditions limit the practical ability of community-based eye screening, especially in rural areas. In addition, ocular dilation is time-consuming and carries a remote risk of precipitating acute angle closure attack, especially in eyes with pre-existing narrow angles. For community-based automated nuclear cataract grading to be scalable and accessible, it should be performed with portable devices under physiologic conditions. However, the ability to autonomously grade nuclear cataracts without dilation and under heterogeneous conditions is still wanting.

**Figure 1:**
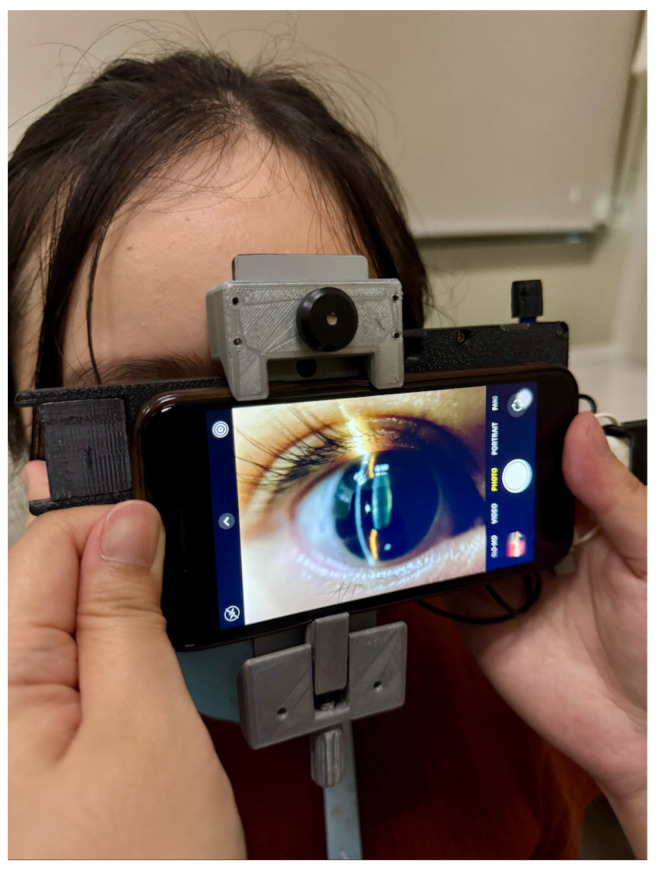
Photograph of the prototype device set-up and slit lamp image capture in a study participant. This photo was taken in a bright room for illustration purposes; in the study, photos were captured in a dim room to simulate mesopic conditions.

In this pilot study, we describe the results of using deep learning to grade nuclear cataracts using a portable smartphone-based portable slit lamp (Figure 1), with and without dilation. We aim to determine the feasibility of grading nuclear cataracts in undilated eyes, which may serve as the basis to facilitate large-scale population-based screening.

## 2. Materials and Methods

### 2.1. Patient Recruitment and Image Capture

This was a prospective, single-institution, cross-sectional study conducted between 28 April 2022 and 11 July 2023 (both dates inclusive). Consecutive eligible patients were recruited from the tertiary eye clinic of the National University Hospital, Singapore. The inclusion criteria were as follows:

- Willing and able to participate in study, with mental capacity to consent;
- At least 40 years old;
- Having not had prior intraocular surgery or laser procedures to the eye, including laser refractive surgery;
- Fit enough to keep the eyes open for adequate image acquisition;
- No evidence of active intraocular inflammation;
- Not having concurrent external or anterior segment pathologies (e.g., corneal opacities, significant blepharoptosis), which would obscure photo-taking of the eye.

The research followed the tenets of the Declaration of Helsinki and was approved by the institutional domain-specific review board. Informed consent was obtained from all subjects after an explanation of the purpose and possible consequences of the study. If both eyes were eligible, both were included in the study.

### 2.2. Image Capture Protocol

**Figure 2:**
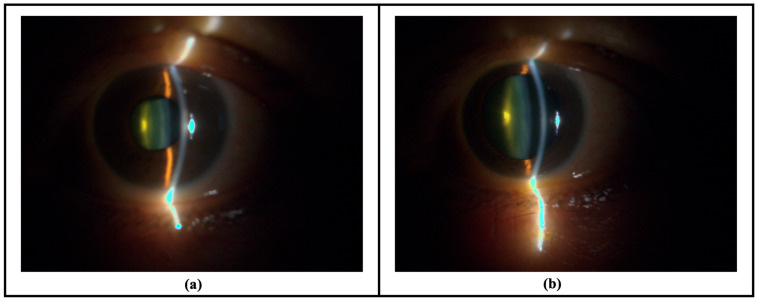
Sample of slit lamp photographs taken with a smartphone camera with the prototype Device in: (**a**) an undilated eye; and (**b**) the same eye after dilation.

After informed consent, all patients underwent a comprehensive ophthalmic examination by a single ophthalmologist (D.C.). Slit lamp photos were then captured with a portable device (the ‘Device’, Figure 1, also described previously elsewhere [14]). The technique for image capture was modified from a previous publication [15]. Briefly, a second-generation iPhone SE (Apple, Cupertino, CA, USA) with the Device was used for anterior segment photos in a dimmed room to simulate mesopic conditions. The Device comprises a light-emitting diode (LED) module fitted behind an optical slit and achromatic condensing lenses to produce an incident light of 45°, with the beam measuring 1 mm wide by 15 mm tall. The Device is held ‘en face’ at a working distance of approximately 2 cm from the eye, with the slit beam originating temporally and was aimed at the pupil center to produce a cross-sectional image of the crystalline lens (Figure 2). Five images were captured of each eye. The same slit lamp photo capture sequence was repeated after pharmacological dilation with one drop of tropicamide 1% (1% Mydriacyl^®^, Alcon Inc., Fort Worth, TX, USA) into the inferior fornix.

To reflect real-world usage of smartphone applications, we captured the images using the default camera application (“Camera”) on the smartphone on automatic settings. We saved the images as digital negative (DNG) files, with 4032 × 3024 resolution, to preserve maximum image fidelity and minimize software post processing. The full uncompressed DNG images were used for automatic cataract analysis.

Scheimpflug imaging with Pentacam^®^ (Oculus, Inc., Arlington, WA, USA) was performed on the dilated eye. Pentacam grading was performed using Pentacam Nuclear Staging (PNS) software (Version 1.21r24). The PNS classification evaluates the optical density of a central, 4 mm three-dimensional reference block of the lens by analyzing the backward scatter when the eye is dilated, grading it from 0 to 5 [16]. PNS classification has been demonstrated to accurately estimate nuclear cataracts [17,18,19]; higher PNS scores are associated with the increased energy used during cataract surgery [20,21]. As a pilot study to explore the feasibility of using a portable device to identify cataracts that may be visually significant, we classified the attained PNS scores into two groups: Group 1 (PNS < 2), and Group 2 (PNS ≥ 2). The latter is correlated with significantly greater cataract density and energy used during phacoemulsification [20].

### 2.3. Deep Learning Technique

**Figure 3:**
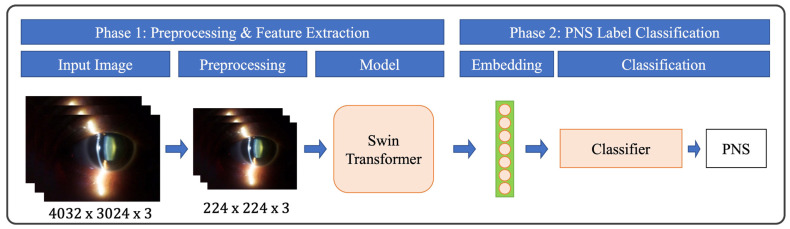
The schematic flow of the adopted deep learning model. PNS, Pentacam nuclear staging.

With our preliminary dataset, we have implemented a transformer-based network [22] for automated cataract analysis (Figure 3). The Swin transformer [23] represents a cutting-edge architecture, employing hierarchical self-attention mechanisms that efficiently capture both local and global features. We selected Swin transformer as the primary backbone because it is a widely validated state-of-the-art architecture for image classification and medical image analysis. By partitioning the input image into non-overlapping patches and progressively merging neighboring patches, the network performs multi-scale feature extraction, making it well-suited for the nuanced demands of medical image analysis, where both fine-grained details and broader structural patterns are critical [24,25]. Unlike traditional convolutional networks that depend on fixed-size kernels, the transformer-based approach adaptively models relationships between image patches. This flexibility allows the network to effectively capture varying degrees of cataract severity through both local and global image features, providing a promising tool for analyzing ocular images from portable smartphone-based slit lamp prototypes.

In this study, we trained the Swin transformer (Swin small variant pretrained on ImageNet-1K) to project images into a high-dimensional embedding space, leveraging the output from the global average pooling layer following the stacked self-attention blocks as the feature representation for each image. The model was optimized using cross-entropy loss, where prediction probabilities were computed via a SoftMax function applied to the class logits. Stochastic gradient descent was employed for optimization. An L2 regularization term (weight decay) was applied during optimization to improve generalization and prevent overfitting. The model was trained end-to-end without freezing backbone layers.

During inference, image labels were predicted by projecting the input into the embedding space and assigning the class label corresponding to the highest predicted probability. All models were implemented using the Apache SINGA platform [26], which integrates with the PyTorch 1.8 backend for model training and distributed optimization, while MLCask [27], an efficient pipeline management system, was employed to handle versioning and management of our deep learning pipelines.

### 2.4. Experiment Setup for Deep Learning Model

**Figure 4:**
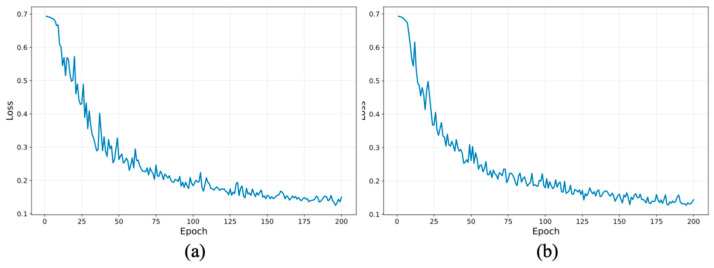
Training loss curves of the Swin transformer model over 200 epochs: (**a**) loss curves for undilated eye images; and (**b**) Loss curves for dilated eye images. Both plots demonstrate stable optimization and convergence prior to completion of the full training schedule.

The input images were captured with a native resolution of 4032 × 3024. Before being fed into the model, all images were resized to 224 × 224 pixels using bicubic interpolation to maintain consistent input dimensions and manageable computational cost. Although the model operates on the resized inputs, acquiring images at a high resolution remains beneficial during data collection, as it helps preserve overall image quality, reduces the impact of sensor noise, and provides reliable source images for subsequent preprocessing, and annotation. The resized images were then normalized using the standard ImageNet mean and standard deviation to match the input distribution expected by the pretrained model. During training, we adopted a comprehensive data augmentation pipeline via the create_transform function from the TIMM package. Input images were resized to 224 × 224 pixels and subjected to color jittering with a factor of 0.4, the ‘rand-m9-mstd0.5-inc1’ AutoAugment policy to enhance sample diversity, and random erasing (pixel-level mode, probability 0.25, with a single erasing count) to improve generalization. Bicubic interpolation was consistently used for image rescaling. The model was trained for 200 epochs using the AdamW optimizer (weight decay = 0.05) with an initial learning rate of 9.735 × 10^−5^ (cosine learning rate scheduling was applied). Training was performed using distributed data parallelism across three NVIDIA RTX 2080 Ti GPUs, with a batch size of 32 per GPU and gradient accumulation over 2 steps, resulting in an effective global batch size of 192. Early convergence was observed before completion of 200 epochs. All experiments were conducted on a workstation equipped with 64 GB RAM and an Intel^®^ Xeon^®^ W-2133 CPU @ 3.60 GHz, with a total training time of approximately 30 min. Training loss curves (Figure 4) demonstrate that convergence was achieved before completion of 200 epochs, supporting the adequacy of this training schedule.

## 3. Results

### 3.1. Patient Characteristics

**Figure 5:**
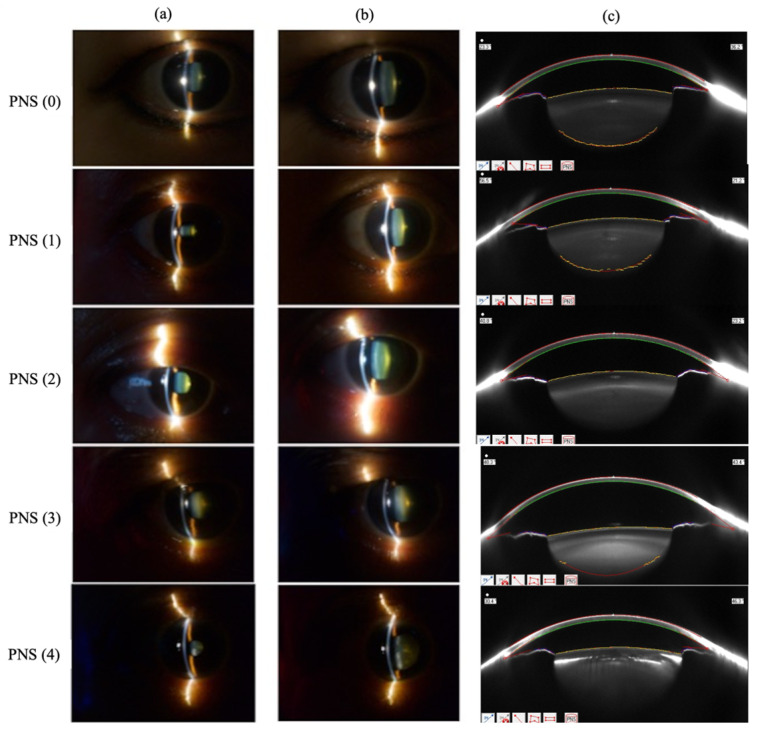
Slit lamp photographs taken: (**a**) before; and (**b**) after dilation with the (**c**) corresponding Pentacam nuclear staging (PNS) scores. A more positive PNS score indicates higher cataract density.

In total, 198 eyes of 99 subjects were included in this study. The mean age of included subjects was 65.3 ± 10.4 years (range, 41.0 to 88.0 years). Of the 198 eyes included in the study, the average PNS score was 1.57 ± 0.81 (range, 0 to 4). Figure 5 illustrates the clinical slit lamp photographs and corresponding PNS scores.

### 3.2. Deep Learning Model on Automated Cataract Analysis

After excluding 80 images (4.0%) deemed of poor quality, a total of 1900 images (950 dilated, 950 undilated) of 198 eyes were included. Table 1 shows the detailed data statistics (note the undilated images and dilated images share the same PNS statistics). We randomly split the dataset at the patient level into a ratio at around 9:1:2 for training, validation, and testing, respectively, following standard machine learning practice. The split ratio was applied to the overall dataset rather than enforced separately for each class. Because the numbers of Group 1 and Group 2 samples were not equal and patient-level partitioning was preserved to prevent data leakage, per class sample counts in each subset do not exactly follow the 9:1:2 proportion. We used the training set for learning the model and validation set to select the hyper-parameters of our deep learning method. Finally, we report the performance of our model on the test set.

Table 2 and Table 3 show the experiment results of our developed deep learning model on automated cataract analysis in undilated and dilated eyes, respectively. For evaluation, we used class average accuracy to measure the performance of the deep learning model. With the preliminary dataset, the deep learning model achieved an average accuracy of 81.25% for undilated images and 74.38% for dilated images. Although the overall accuracy was moderate, it is important to note that images were acquired using a portable smartphone-based slit lamp under heterogeneous real-world conditions, including undilated eyes. This setting is substantially more challenging than standardized, clinic-based imaging protocols. As a pilot feasibility study, these results demonstrate the practical potential of AI-assisted cataract grading in portable screening scenarios. The confusion matrices also reveal asymmetric error patterns. In both undilated and dilated eye experiments, false positives (i.e., Group 1 predicted as Group 2) occurred more frequently than false negatives. Consequently, the model demonstrates high sensitivity but relatively lower specificity. From a clinical screening perspective, this asymmetry may be acceptable because missing clinically significant cataracts (false negatives) may delay diagnosis and referral, whereas false positives mainly result in additional confirmatory examination.

In particular, performance was slightly lower in dilated eyes compared to undilated eyes. While this may initially seem counterintuitive given that clinical ground truth is established using dilated examinations, it likely stems from differences in how deep learning models process visual information compared to human clinicians. Clinicians rely on dilation to evaluate the entire crystalline lens, particularly peripheral cortical features. For the Swin transformer, however, the undilated pupil effectively acts as a natural anatomical crop. The constricted iris masks out peripheral regions, inadvertently guiding the model’s attention directly to the central lens, which contains consistent, high-yield diagnostic features of opacity. Conversely, pharmacological dilation exposes a significantly larger area of the anterior segment. This introduces complex peripheral light scattering, iris shadows, and a wider surface area for specular reflections. Because the model was trained on whole-image classification without explicit pixel-level segmentation to restrict its focus, this newly exposed peripheral area acts as visual noise. The model may become distracted by these peripheral optical artifacts in dilated images, leading to greater feature variability and slightly lower classification accuracy compared to the naturally constrained field of view in undilated eyes.

In addition, Table 4 presents the model performance on both undilated and dilated eye images, reporting predictive uncertainty (measured via entropy) alongside standard classification metrics for each class label. For undilated eyes, the model achieved a high overall accuracy of 81.25%. Specifically, for Group 2, it attained perfect recall (100%) and a precision of 72.73%, yielding an F1 score of 84.21%. For Group 1, the model demonstrated 100% precision, 62.50% recall, and an F1 score of 76.92%. In the case of dilated eyes, performance declined moderately, with an overall accuracy of 74.38%. Notably, the predictive entropy was 0.3163 for undilated and 0.3376 for dilated eyes, indicating generally low uncertainty in both cases, although slightly higher ambiguity in the dilated condition, which is consistent with its comparatively reduced predictive performance. However, entropy was not used for threshold-based rejection or selective prediction in this pilot study, and, therefore, should be interpreted as a descriptive confidence measure rather than a validated reliability guarantee. Future work will investigate uncertainty calibration and selective prediction strategies to enhance clinical robustness.

Beyond overall accuracy, Table 5 presents the sensitivity, specificity, receiver operating characteristic–area under the curve (ROC–AUC), and calibration metrics (Brier score and expected calibration error with 10 bins). The ROC–AUC was 84.30% (undilated) and 76.55% (dilated), indicating good discriminative ability. Calibration performance was assessed using the Brier score (16.39 and 20.15) and expected calibration error (ECE-BIN10: 15.81 and 17.78), demonstrating reasonable probability calibration in this pilot setting.

### 3.3. Comparison with Other Baseline Architectures

To strengthen the validity of our findings, in Table 6, we compared the Swin transformer backbone with three widely used image classification architectures: ResNet50 [28], EfficientNet [29], and Vision Transformer (ViT-B/16) [30]. All baseline models were trained and evaluated under the same data split, preprocessing pipeline, and optimization settings to ensure fair comparison.

For undilated eyes, ViT-B/16 achieved the highest overall accuracy (80.26%), followed by EfficientNet (79.65%) and ResNet50 (78.60%). Notably, ViT demonstrated a more balanced performance between the two classes, achieving a Group 2 F1 score of 58.82%, compared to 34.09% for EfficientNet and 32.97% for ResNet50. Both EfficientNet and ResNet50 showed high recall for Group 1 (>92%), but substantially lower recall for Group 2 (25%), indicating a bias toward the majority class.

For dilated eyes, ViT-B/16 again achieved the highest overall accuracy (82.11%), followed by ResNet50 (72.42%) and EfficientNet (69.82%). ResNet50 showed relatively strong performance for Group 2 in dilated eyes (F1 = 65.26%), while EfficientNet demonstrated lower precision for Group 2 (34.88%). ViT achieved high Group 1 recall (97.33%) but comparatively lower recall for Group 2 (25%), suggesting potential class imbalance sensitivity.

Overall, transformer-based architectures (ViT and Swin transformer) demonstrated competitive or superior performance compared with conventional convolutional networks (EfficientNet and ResNet50), particularly in maintaining better balance across class-specific metrics. These results support the suitability of transformer-based backbones for cataract severity classification in portable slit lamp imaging scenarios.

### 3.4. Heat Maps

**Figure 6:**
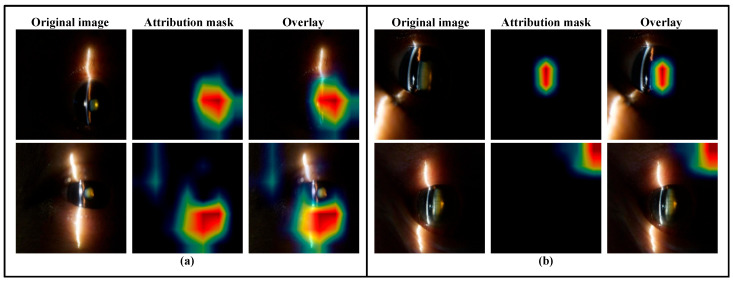
Heat map demonstrating the original image, attribution mask, and overlay of the attribution mask over the image with integrated gradient method: (**a**) an undilated eye with correct attribution on the top row, and incorrect attribution on the bottom row; and (**b**) a dilated eye with correct attribution on the top row, and incorrect attribution on the bottom row.

Based on the transformer prototypical network, heat maps were generated to visualize regions contributing to cataract severity prediction. The integrated gradients method [31] was adopted for visualization, which is an attribution method for deep networks. As shown in Figure 6, in correctly predicted cases, the attribution maps generally highlight the central crystalline lens region corresponding to cataract opacity. In contrast, misclassified examples tend to exhibit more diffuse or misplaced attention patterns, often associated with specular reflections or peripheral illumination artifacts.

Because pixel-level annotations of cataract regions were not available in this pilot dataset, automated spatial localization metrics could not be computed. To account for this quantitatively, we conducted a manual review of the attribution maps for the test set. Among correctly predicted cases, all the attribution maps accurately localized the primary attention on the central crystalline lens. Conversely, in misclassified examples, the model’s attention was frequently misplaced: a total of 57.75% of these errors were driven by heavy focus on specular corneal reflections, 35.21% were misdirected by peripheral illumination artifacts, and 7.04% exhibited a highly diffuse attention pattern lacking any distinct focal region. Therefore, while the attribution maps are presented primarily as qualitative visualizations, this semi-quantitative breakdown illustrates that the model generally learns the correct anatomical features for accurate prediction, whereas misclassifications are strongly linked to identifiable imaging artifacts.

## 4. Discussion

In this pilot study, we describe a novel method to grade nuclear cataracts using slit lamp images taken from a smartphone-based portable slit lamp device in undilated eyes. Our preliminary results suggest that AI was able to grade cataracts in both dilated and undilated eyes, although the accuracy of AI on the undilated eyes was higher. The heat maps generated based on attribution masks provide preliminary evidence that the integrated gradient could correctly identify the anatomical areas of concern.

The benefit of having an automated digital grading for cataracts is clear. Cataracts are a universal age-related opacification of the crystalline lens that affect everyone from around 60 years old. A digital cataract grading system was shown to be more cost-effective than human grading as early as 1999 [32]. However, it is not fully implemented; some challenges include the routine need for dilation and the strict requirements for image standardization. While severe cataracts could be diagnosed with low-cost screening methods with a penlight and crude visual acuity assessment, a cost-effective solution to identify moderate yet visually significant cataracts is still lacking.

Over the past decade, there has been increasing interest in using deep learning to grade nuclear cataracts automatically (Table 7). These studies utilized standardized slit lamp images from existing databases, such as the Singapore Malay Eye Study [13], the Age-Related Eye Disease Study (AREDS) [33], or retrospective datasets of clinical slit lamp images compared against different clinical standards. However, while these studies report impressive performances, they were all trained on existing databases of dilated slit lamp photographs taken under strict lighting conditions, which significantly limit their applicability for use as a community-based screening tool where dedicated slit lamp cameras and exacting lighting requirements are not possible. This set-up—in particular, ocular dilation—is not routinely performed outside of eye clinics. Conversely, alternative approaches for community-based smartphone cataract screening using AI do not yet incorporate slit lamp imaging [34,35,36], even though slit lamp-based photographs are widely regarded as the superior method for assessing nuclear cataracts [37].

To bridge this gap, our work is the first to report automated cataract grading using a portable smartphone slit lamp set-up. In addition, our study was performed on undilated eyes under real-world, pragmatic, imaging conditions. While the deep learning backbone itself is not novel, the innovation of this study lies in translating state-of-the-art AI methods into a portable, smartphone-based slit lamp platform and evaluating its feasibility in a non-standardized, undilated clinical setting. If fully deployed, this approach could help to overcome key practical barriers to community-based cataract screening and expand access to early detection. Another strength of our study is that a standardized protocol was used for image capture; the ground truth used in this study (PNS) was shown to be an objective and reproducible way to grade cataracts [18]. Standardized grading scales, such as the LOCS III, on the other hand, have been reported to have suboptimal reproducibility [33,43,44]. A strength of our AI approach lies in its enhanced representation capability, derived from the attention mechanism to effectively model complex patterns specific to the medical domain [24,25]. Recent studies [45,46] have further demonstrated the effectiveness of transformer-based architectures and deep learning models in ophthalmic imaging and medical image classification tasks under real-world conditions, supporting the methodological choice and performance level observed in our work.

This preliminary study is limited by the small sample size of the images tested; it is likely that the initial developed model has issues with overfitting. In addition, as this study adopts established backbone architectures without introducing new architectural modules, systematic ablation experiments were not performed in the current work. Such analyses will be incorporated in future studies to better quantify the contribution of different model components and training strategies. Likewise, due to the small sample size, the heat maps of typical images were overlaid on other images, leading to issues with attribution mask allocation (Figure 6, bottom row). Larger, multi-center studies with broader demographic representation will be necessary to confirm the robustness and external validity of these findings. Another limitation of our study is the relatively mild cataracts in the captured dataset—all the cataracts identified were less than PNS 5, with the majority being graded as 0 to 1 (88 of 198 eyes, 44.4%). The accuracy of our deep learning model is also lower compared to alternative algorithms published previously [40,47]. However, ours is an initial proof-of-concept study to establish the feasibility of evaluating cataract density in undilated eyes captured on mobile devices. Our initial results are encouraging.

In conclusion, we have demonstrated preliminary results indicating that deep learning could be used to grade cataracts with slit lamp images taken from undilated eyes. Further work is underway to validate this technique in a larger and more diverse group of eyes with cataracts.

## Figures and Tables

**Table 1:** Data statistics of collected undilated dataset.

PNS Label	Group 1 (PNS Score < 2)	Group 2 (PNS Score ≥ 2)	Total
Training	320	390	710
Validation	40	40	80
Test	80	80	160
Total	440	510	950

PNS, Pentacam nuclear staging.

**Table 2:** Performance of developed deep learning model on automated cataract analysis in undilated eyes.

		**PNS Label**		**Average Accuracy** **(%)**
		**Group 1 (PNS Score < 2)**	**Group 2 (PNS Score ≥ 2)**	
Predicted Label	Group 1 (PNS score < 2)	50	0	
	Group 2 (PNS score ≥ 2)	30	80	
Model Accuracy (%)		62.50	100.00	81.25

PNS, Pentacam nuclear staging.

**Table 3:** Performance of developed deep learning model on automated cataract analysis in dilated eyes.

		**PNS Label**		**Average Accuracy (%)**
		**Group 1 (PNS Score < 2)**	**Group 2 (PNS Score ≥ 2)**	
Predicted Label	Group 1 (PNS Score < 2)	44	5	
	Group 2 (PNS score ≥ 2)	36	75	
Model Accuracy (%)		55.00	93.75	74.38

PNS, Pentacam nuclear staging.

**Table 4:** Detailed metrics of developed deep learning model on automated cataract analysis.

			Group 2 (PNS Score ≥ 2)			Group 1 (PNS Score < 2)		
	Uncertainty	Accuracy (%)	Precision (%)	Recall (%)	F1 (%)	Precision (%)	Recall (%)	F1 (%)
Undilated Eyes	0.3163	81.25	72.73	100.00	84.21	100	62.50	76.92
Dilated Eyes	0.3376	74.38	67.57	93.75	78.53	89.80	55.00	68.22

PNS, Pentacam nuclear staging.

**Table 5:** Discrimination and calibration performance on automated cataract analysis.

	Sensitivity (%)	Specificity (%)	ROC–AUC (%)	Brier Score (%)	ECE (BIN10) (%)
Undilated Eyes	100.00	62.50	84.30	16.39	15.81
Dilated Eyes	93.75	55.00	76.55	20.15	17.78

ECE (BIN10), expected calibration error with 10 bins); ROC–AUC, receiver operating characteristic–area under the curve.

**Table 6:** Performance comparison of baseline architectures (EfficientNet, ResNet50, and ViT-B/16) on automated cataract analysis.

**ResNet50**							
		Group 2 (PNS score ≥ 2)			Group 1 (PNS Score < 2)		
	Accuracy (%)	Precision (%)	Recall (%)	F1 (%)	Precision (%)	Recall (%)	F1 (%)
Undilated Eyes	78.60	48.39	25.00	32.97	82.28	92.89	87.27
Dilated Eyes	72.42	88.57	51.67	65.26	78.40	81.22	79.79
**EfficientNet**							
		Group 2 (PNS score ≥ 2)			Group 1 (PNS Score < 2)		
	Accuracy (%)	Precision (%)	Recall (%)	F1 (%)	Precision (%)	Recall (%)	F1 (%)
Undilated Eyes	79.65	53.57	25.00	34.09	82.49	94.22	87.97
Dilated Eyes	69.82	34.88	50.00	41.10	84.92	75.11	79.72
**ViT-B/16**							
		Group 2 (PNS score ≥ 2)			Group 1 (PNS Score < 2)		
	Accuracy (%)	Precision (%)	Recall (%)	F1 (%)	Precision (%)	Recall (%)	F1 (%)
Undilated Eyes	80.26	71.43	50.00	58.82	87.65	94.67	91.03
Dilated Eyes	82.11	71.43	25.00	37.04	82.95	97.33	89.57

PNS, Pentacam nuclear staging.

**Table 7:** Literature review on automatic nuclear cataract grading using slit lamp images from 2015 onwards.

Author (Year)	Dataset	Clinical Reference	Performance
Gao et al. (2015) [12]	5378 slit lamp images of dilated eyes	Wisconsin cataract grading system [4]	70.7% exact agreement ratio, 88.4% decimal grading error ≤ 1.0
Wu et al. (2019) [38]	37,638 slit lamp images of dilated and undilated eyes	LOCS II [2] (sub-divided into severe or mild)	AUC > 91%
Son et al. (2022) [39]	1355 slit lamp images of dilated eyes	LOCS III [3]	AUC 95.7%
Lu et al. (2022) [40]	1039 slit lamp images of dilated eyes	LOCS III [3] (sub-divided into severe or mild)	AUC between 97.7% and 98.3%
Keenan et al. (2022) [41]	6333 slit lamp images of dilated eyes	AREDS system [42]	MSE = 0.23
Goh et al. (2025) [37]	12,067 slit lamp images of dilated eyes	Wisconsin cataract grading system [4]	AUC between 92.3% to 93.4%

AUC, area under the receiver operating characteristic curve; LOCS, Lens Opacities Classification System; MSE, mean squared error.

## Data Availability

The data presented in this study are available on request from the corresponding author. The data are not publicly available due to institutional restrictions.

## References

[1] Lee C.M., Afshari N.A. (2017). The Global State of Cataract Blindness. Curr. Opin. Ophthalmol..

[2] Chylack L.T., Leske M.C., McCarthy D., Khu P., Kashiwagi T., Sperduto R. (1989). Lens Opacities Classification System II (LOCS II). Arch. Ophthalmol..

[3] Chylack L.T., Wolfe J.K., Singer D.M., Leske M.C., Bullimore M.A., Bailey I.L., Friend J., McCarthy D., Wu S.Y. (1993). The Lens Opacities Classification System III. The Longitudinal Study of Cataract Study Group. Arch. Ophthalmol..

[4] Klein B.E.K., Klein R., Linton K.L.P., Magli Y.L., Neider M.W. (1990). Assessment of Cataracts from Photographs in the Beaver Dam Eye Study. Ophthalmology.

[5] Sparrow J.M., Bron A.J., Brown N.A., Ayliffe W., Hill A.R. (1986). The Oxford Clinical Cataract Classification and Grading System. Int. Ophthalmol..

[6] Wong W.L., Li X., Li J., Cheng C.-Y., Lamoureux E.L., Wang J.J., Cheung C.Y., Wong T.Y. (2013). Cataract Conversion Assessment Using Lens Opacity Classification System III and Wisconsin Cataract Grading System. Investig. Ophthalmol. Vis. Sci..

[7] Xu Y., Gao X., Lin S., Wong D.W.K., Liu J., Xu D., Cheng C.-Y., Cheung C.Y., Wong T.Y. (2013). Automatic Grading of Nuclear Cataracts from Slit-Lamp Lens Images Using Group Sparsity Regression. Med. Image Comput. Comput. Assist. Interv..

[8] Huang W., Chan K.L., Li H., Lim J.H., Liu J., Wong T.Y. (2011). A Computer Assisted Method for Nuclear Cataract Grading from Slit-Lamp Images Using Ranking. IEEE Trans. Med. Imaging.

[9] Cheung C.Y., Li H., Lamoureux E.L., Mitchell P., Wang J.J., Tan A.G., Johari L.K., Liu J., Lim J.H., Aung T. (2011). Validity of a New Computer-Aided Diagnosis Imaging Program to Quantify Nuclear Cataract from Slit-Lamp Photographs. Investig. Ophthalmol. Vis. Sci..

[10] Li H., Lim J.H., Liu J., Wong D.W.K., Tan N.M., Lu S., Zhang Z., Wong T.Y. (2009). An Automatic Diagnosis System of Nuclear Cataract Using Slit-Lamp Images. Annu. Int. Conf. IEEE Eng. Med. Biol. Soc..

[11] Ignatowicz A.A., Marciniak T., Marciniak E. (2025). AI-Powered Mobile App for Nuclear Cataract Detection. Sensors.

[12] Gao X., Lin S., Wong T.Y. (2015). Automatic Feature Learning to Grade Nuclear Cataracts Based on Deep Learning. IEEE Trans. Biomed. Eng..

[13] Foong A.W.P., Saw S.-M., Loo J.-L., Shen S., Loon S.-C., Rosman M., Aung T., Tan D.T.H., Tai E.S., Wong T.Y. (2007). Rationale and Methodology for a Population-Based Study of Eye Diseases in Malay People: The Singapore Malay Eye Study (SiMES). Ophthalmic Epidemiol..

[14] Yu Y., Chen D., Tan C.W.T., Cheng C.Y., Yong S.S. Mobile Eye-Imaging Device for Detecting Eye Pathologies 2020. https://patents.google.com/patent/WO2020060486A1/en?oq=WO2020%2f060486+A1.

[15] Chen D., Ho Y., Sasa Y., Lee J., Yen C.C., Tan C. (2021). Machine Learning-Guided Prediction of Central Anterior Chamber Depth Using Slit Lamp Images from a Portable Smartphone Device. Biosensors.

[16] Ambrosio R. Oculus Pentacam Interpretation Guide.

[17] Panthier C., de Wazieres A., Rouger H., Moran S., Saad A., Gatinel D. (2019). Average Lens Density Quantification with Swept-Source Optical Coherence Tomography: Optimized, Automated Cataract Grading Technique. J. Cataract. Refract. Surg..

[18] Pan A.-P., Wang Q.-M., Huang F., Huang J.-H., Bao F.-J., Yu A.-Y. (2015). Correlation Among Lens Opacities Classification System III Grading, Visual Function Index-14, Pentacam Nucleus Staging, and Objective Scatter Index for Cataract Assessment. Am. J. Ophthalmol..

[19] Pei X., Bao Y., Chen Y., Li X. (2008). Correlation of Lens Density Measured Using the Pentacam Scheimpflug System with the Lens Opacities Classification System III Grading Score and Visual Acuity in Age-Related Nuclear Cataract. Br. J. Ophthalmol..

[20] Mayer W.J., Klaproth O.K., Hengerer F.H., Kohnen T. (2014). Impact of Crystalline Lens Opacification on Effective Phacoemulsification Time in Femtosecond Laser-Assisted Cataract Surgery. Am. J. Ophthalmol..

[21] Nixon D.R. (2010). Preoperative Cataract Grading by Scheimpflug Imaging and Effect on Operative Fluidics and Phacoemulsification Energy. J. Cataract. Refract. Surg..

[22] Vaswani A., Shazeer N., Parmar N., Uszkoreit J., Jones L., Gomez A.N., Kaiser Ł., Polosukhin I. (2017). Attention Is All You Need. Proceedings of the 31st International Conference on Neural Information Processing Systems, Long Beach, CA, USA, 4–9 December 2017.

[23] Liu Z., Lin Y., Cao Y., Hu H., Wei Y., Zhang Z., Lin S., Guo B. (2021). Swin Transformer: Hierarchical Vision Transformer Using Shifted Windows. 2021 IEEE/CVF International Conference on Computer Vision (ICCV).

[24] Tang Y., Yang D., Li W., Roth H.R., Landman B., Xu D., Nath V., Hatamizadeh A. (2022). Self-Supervised Pre-Training of Swin Transformers for 3D Medical Image Analysis. 2022 IEEE/CVF Conference on Computer Vision and Pattern Recognition (CVPR).

[25] Cao H., Wang Y., Chen J., Jiang D., Zhang X., Tian Q., Wang M., Karlinsky L., Michaeli T., Nishino K. (2023). Swin-Unet: Unet-Like Pure Transformer for Medical Image Segmentation.

[26] Ooi B.C., Tan K.-L., Wang S., Wang W., Cai Q., Chen G., Gao J., Luo Z., Tung A.K.H., Wang Y. (2015). SINGA: A Distributed Deep Learning Platform. Proceedings of the 23rd ACM International Conference on Multimedia, Brisbane, Australia, 26–30 October 2015.

[27] Luo Z., Yeung S.H., Zhang M., Zheng K., Zhu L., Chen G., Fan F., Lin Q., Ngiam K.Y., Chin Ooi B. (2021). MLCask: Efficient Management of Component Evolution in Collaborative Data Analytics Pipelines. Proceedings of the 2021 IEEE 37th International Conference on Data Engineering (ICDE).

[28] He K., Zhang X., Ren S., Sun J. (2016). Deep Residual Learning for Image Recognition. Proceedings of the 2016 IEEE Conference on Computer Vision and Pattern Recognition (CVPR).

[29] Tan M., Le Q.V. EfficientNet: Rethinking Model Scaling for Convolutional Neural Networks. Proceedings of the 36th International Conference on Machine Learning.

[30] Dosovitskiy A., Beyer L., Kolesnikov A., Weissenborn D., Zhai X., Unterthiner T., Dehghani M., Minderer M., Heigold G., Gelly S. (2021). An Image Is Worth 16 × 16 Words: Transformers for Image Recognition at Scale. arXiv.

[31] Selvaraju R.R., Cogswell M., Das A., Vedantam R., Parikh D., Batra D. (2017). Grad-CAM: Visual Explanations from Deep Networks via Gradient-Based Localization. Proceedings of the 2017 IEEE International Conference on Computer Vision (ICCV), Venice, Italy, 22–29 October 2017.

[32] Dimock J., Robman L.D., McCarty C.A., Taylor H.R. (1999). Cost-Effectiveness of Digital Cataract Assessment. Aust. N. Zealand J. Ophthalmol..

[33] Chew E.Y., Kim J., Sperduto R.D., Datiles M.B., Coleman H.R., Thompson D.J.S., Milton R.C., Clayton J.A., Hubbard L.D., Danis R.P. (2010). Evaluation of the Age-Related Eye Disease Study Clinical Lens Grading System AREDS Report No. 31. Ophthalmology.

[34] Ganokratanaa T., Ketcham M., Pramkeaw P. (2023). Advancements in Cataract Detection: The Systematic Development of LeNet-Convolutional Neural Network Models. J. Imaging.

[35] Pathak S., Raj R., Singh K., Verma P.K., Kumar B. (2022). Development of Portable and Robust Cataract Detection and Grading System by Analyzing Multiple Texture Features for Tele-Ophthalmology. Multimed. Tools Appl..

[36] Janti S.S., Saluja R., Tiwari N., Kolavai R.R., Mali K., Arora A.J., Johar A., Sahoo D.P., Sahithi E. (2024). Evaluation of the Clinical Impact of a Smartphone Application for Cataract Detection. Cureus.

[37] Goh J.H.L., Lei X., Chee M.-L., Qian Y., Yu M., Rim T.H., Nusinovici S., Chen D.Z., Koh K.H., Yew S.M.E. (2025). Multi-Comparison of Different Ocular Imaging Modality-Based Deep Learning Models for Visually Significant Cataract Detection. Ophthalmol. Sci..

[38] Wu X., Huang Y., Liu Z., Lai W., Long E., Zhang K., Jiang J., Lin D., Chen K., Yu T. (2019). Universal Artificial Intelligence Platform for Collaborative Management of Cataracts. Br. J. Ophthalmol..

[39] Son K.Y., Ko J., Kim E., Lee S.Y., Kim M.-J., Han J., Shin E., Chung T.-Y., Lim D.H. (2022). Deep Learning-Based Cataract Detection and Grading from Slit-Lamp and Retro-Illumination Photographs: Model Development and Validation Study. Ophthalmol. Sci..

[40] Lu Q., Wei L., He W., Zhang K., Wang J., Zhang Y., Rong X., Zhao Z., Cai L., He X. (2022). Lens Opacities Classification System III-Based Artificial Intelligence Program for Automatic Cataract Grading. J. Cataract. Refract. Surg..

[41] Keenan T.D.L., Chen Q., Agrón E., Tham Y.-C., Goh J.H.L., Lei X., Ng Y.P., Liu Y., Xu X., Cheng C.-Y. (2022). DeepLensNet: Deep Learning Automated Diagnosis and Quantitative Classification of Cataract Type and Severity. Ophthalmology.

[42] The Age-Related Eye Disease Study Research Group (2001). The Age-Related Eye Disease Study (AREDS) System for Classifying Cataracts from Photographs: AREDS Report No. 4. Am. J. Ophthalmol..

[43] Gali H.E., Sella R., Afshari N.A. (2019). Cataract Grading Systems: A Review of Past and Present. Curr. Opin. Ophthalmol..

[44] Tan A.C.S., Wang J.J., Lamoureux E.L., Wong W., Mitchell P., Li J., Tan A.G., Wong T.Y. (2011). Cataract Prevalence Varies Substantially with Assessment Systems: Comparison of Clinical and Photographic Grading in a Population-Based Study. Ophthalmic Epidemiol..

[45] Wan Z., Zhang J., Wang Y., Lin H., Wang Y., Mi Z., Yang X., Fu X., Wang H. (2025). Eye-Based Emotion Recognition via Event-Driven Sparse Transformers. Proceedings of the 33rd ACM International Conference on Multimedia, Dublin, Ireland, 27–31 October 2025.

[46] Iqbal M.A., Kim J., Han I., Kyun Kim S. (2024). Attention-Driven Feature Fusion Integrating Swin Transformer and CNN Models for Improved Ocular Disease Classification. Proceedings of the 2024 International Conference on Engineering and Emerging Technologies (ICEET).

[47] Zhang H., Niu K., Xiong Y., Yang W., He Z., Song H. (2019). Automatic Cataract Grading Methods Based on Deep Learning. Comput. Methods Programs Biomed..

